# Association Between Alcohol Use Disorder and Receipt of Direct-Acting Antiviral Hepatitis C Virus Treatment

**DOI:** 10.1001/jamanetworkopen.2022.46604

**Published:** 2022-12-14

**Authors:** Lamia Y. Haque, David A. Fiellin, Janet P. Tate, Denise Esserman, Debika Bhattacharya, Adeel A. Butt, Stephen Crystal, E. Jennifer Edelman, Adam J. Gordon, Joseph K. Lim, Jeanette M. Tetrault, Emily C. Williams, Kendall Bryant, Emily J. Cartwright, Christopher T. Rentsch, Amy C. Justice, Vincent Lo Re, Kathleen A. McGinnis

**Affiliations:** 1 Department of Internal Medicine, Yale School of Medicine, New Haven, Connecticut; 2 Yale Program in Addiction Medicine, Yale School of Medicine, New Haven, Connecticut; 3 Department of Health Policy and Management, Yale School of Public Health, New Haven, Connecticut; 4 Veterans Affairs Connecticut Health Care System, West Haven; 5 Department of Biostatistics, Yale School of Public Health, New Haven, Connecticut; 6 Department of Internal Medicine, David Geffen School of Medicine, University of California, Los Angeles, Los Angeles; 7 Veterans Affairs Greater Los Angeles Health Care System, Los Angeles, California; 8 Department of Medicine, Weill Cornell Medicine, New York, New York; 9 Department of Population Health Sciences, Weill Cornell Medicine, New York, New York; 10 Veterans Affairs Pittsburgh Healthcare System, Pittsburgh, Pennsylvania; 11 Center for Health Services Research, Rutgers University, New Brunswick, New Jersey; 12 Department of Social and Behavioral Sciences, Yale School of Public Health, New Haven, Connecticut; 13 Informatics, Decision-Enhancement, and Analytic Sciences Center, Veterans Affairs Salt Lake City Health Care System, Salt Lake City, Utah; 14 Program for Addiction Research, Clinical Care, Knowledge, and Advocacy, Division of Epidemiology, Department of Internal Medicine, University of Utah School of Medicine, Salt Lake City; 15 Seattle-Denver Center of Innovation for Veteran-Centered and Value-Driven Care, Veterans Affairs Puget Sound Health Care System, Seattle, Washington; 16 Health Services Research and Development, Veterans Affairs Puget Sound Health Care System, Seattle, Washington; 17 Department of Health Systems and Population Health, University of Washington, Seattle; 18 HIV/AIDS and Alcohol Research Program, National Institute on Alcohol Abuse and Alcoholism, Bethesda, Maryland; 19 Department of Medicine, Emory School of Medicine, Atlanta, Georgia; 20 Veterans Affairs Atlanta Health Care System, Atlanta, Georgia; 21 London School of Hygiene and Tropical Medicine, London, United Kingdom; 22 Division of Infectious Diseases, Department of Medicine, Perelman School of Medicine, Philadelphia, Pennsylvania; 23 Center for Clinical Epidemiology and Biostatistics, Perelman School of Medicine, Philadelphia, Pennsylvania; 24 Department of Biostatistics, Epidemiology, and Informatics, Perelman School of Medicine, Philadelphia, Pennsylvania

## Abstract

**Importance:**

Direct-acting antiviral (DAA) treatment for hepatitis C virus (HCV) infection is associated with lower mortality and is effective in individuals with alcohol use disorder (AUD). However, despite recommendations, patients with AUD may be less likely to receive DAAs.

**Objective:**

To assess the association between alcohol use and receipt of DAA treatment among patients with HCV within the Veterans Health Administration (VHA).

**Design, Setting, and Participants:**

This cohort study included 133 753 patients with HCV born from 1945 to 1965 who had completed the Alcohol Use Disorders Identification Test–Consumption (AUDIT-C) questionnaire and had at least 1 outpatient visit in the VHA from January 1, 2014, through May 31, 2017, with maximal follow-up of 3 years until May 31, 2020; DAA receipt; or death, whichever occurred first.

**Exposures:**

Alcohol use categories generated using responses to the AUDIT-C questionnaire and *International Classification of Diseases, Ninth Revision* and *International Statistical Classification of Diseases and Related Health Problems, Tenth Revision* diagnoses: current AUD, abstinent with AUD history, at-risk drinking, lower-risk drinking, and abstinent without AUD history. Demographic, other clinical, and pharmacy data were also collected.

**Main Outcomes and Measures:**

Associations between alcohol use categories and DAA receipt within 1 and 3 years estimated using Cox proportional hazards regression stratified by calendar year.

**Results:**

Of 133 753 patients (130 103 men [97%]; mean [SD] age, 60.6 [4.5] years; and 73 493 White patients [55%]), 38% had current AUD, 12% were abstinent with a history of AUD, 6% reported at-risk drinking, 14% reported lower-risk drinking, and 30% were abstinent without a history of AUD. Receipt of DAA treatment within 1 year was 7%, 33%, 53%, and 56% for patients entering the cohort in 2014, 2015, 2016, and 2017, respectively. For patients entering in 2014, those with current AUD (hazard ratio [HR], 0.72 [95%, CI, 0.66-0.77]) or who were abstinent with an AUD history (HR, 0.91 [95% CI, 0.84-1.00]) were less likely to receive DAA treatment within 1 year compared with patients with lower-risk drinking. For those entering in 2015-2017, patients with current AUD (HR, 0.75 [95% CI, 0.70-0.81]) and those who were abstinent with an AUD history (HR, 0.76 [95% CI, 0.68-0.86]) were less likely to receive DAA treatment within 1 year compared with patients with lower-risk drinking.

**Conclusions and Relevance:**

This cohort study suggests that individuals with AUD, regardless of abstinence, were less likely to receive DAA treatment. Improved access to DAA treatment for persons with AUD is needed.

## Key Points

**Question:**

What is the association of alcohol use with receipt of direct-acting antiviral (DAA) treatment for hepatitis C virus (HCV) infection?

**Findings:**

In this cohort study of 133 753 adults with HCV infection, those with alcohol use disorder, even if abstinent, were statistically significantly less likely to receive DAA treatment within 1 and 3 years after eligibility compared with those with lower-risk drinking after adjusting for demographic characteristics and comorbidities.

**Meaning:**

This study suggests that, although guidelines recommend DAA treatment for patients with HCV regardless of alcohol use, patients with alcohol use disorder encounter barriers to HCV treatment.

## Teaser

This cohort study of patients within the Veterans Health Administration assesses the association between alcohol use disorder and receipt of direct-acting antiviral treatment for hepatitis C virus (HCV) infection.

## Introduction

Untreated hepatitis C virus (HCV) infection is associated with substantial morbidity and mortality due to cirrhosis, liver cancer, and related complications, especially for individuals with concomitant alcohol-associated liver disease.^1^ Treatment for HCV infection has changed rapidly since 2014 with the introduction of highly efficacious direct-acting antivirals (DAAs).^1,2,3^ Direct-acting antiviral treatment for HCV infection is associated with lower mortality and is effective in individuals with alcohol use disorder (AUD). Patients with HCV and AUD or other substance use disorders (SUDs) have faced barriers to DAA treatment that have limited access to these medications.^4^

There is no known safe level of alcohol use among patients with HCV.^1,5^ Alcohol can accelerate fibrosis and increase the risk of liver-related complications, even after sustained virologic response (SVR) or HCV cure.^6,7^ Despite this risk, unhealthy alcohol use—the range of drinking that includes at-risk use and AUD^8^—is common among patients with HCV.^9,10^ Although clinical trials of DAAs typically excluded patients who drink alcohol,^11^ observational studies have demonstrated high rates of SVR among patients consuming any level of alcohol during DAA treatment.^12^ Clinical guidelines issued by the American Association for the Study of Liver Diseases and the Infectious Diseases Society of America (AASLD-IDSA) have recommended DAA treatment without consideration of alcohol use.^1^

The Veterans Health Administration (VHA) is the largest integrated health care system and provider of HCV treatment in the United States.^13^ Treatment initiatives led by regional multidisciplinary Hepatitis C Innovation Teams support clinicians, including primary care clinicians, specialists, and pharmacists delivering DAA treatment.^14^ Similar to AASLD-IDSA guidelines, VHA guidelines published since 2014 have not excluded patients with AUD from DAA treatment but have emphasized that collaboration with mental health and addiction specialists be considered when offering DAAs.^15,16,17^

Little is known regarding whether alcohol use is a barrier to DAA treatment in clinical practice. Therefore, we sought to evaluate the association between alcohol use and DAA treatment among patients in the VHA with HCV and to examine whether the association has changed over time.

## Methods

### Data Source

We conducted a cohort study using data from the VHA Birth Cohort, an observational study of all individuals born from 1945 to 1965 with at least 1 health care visit at any VHA location from January 1, 2000, to September 30, 2016.^18,19^ We used this data source because persons born from 1945 to 1965 have a 6-fold higher prevalence of HCV compared with all other age groups and have been a major focus of HCV screening efforts by the Centers for Disease Control and Prevention and the VHA.^19,20,21^ The VHA Birth Cohort consists of electronic health record data available through the national Corporate Data Warehouse, a data repository that includes demographic information, medical diagnoses (recorded by *International Classification of Diseases, Ninth Revision* [*ICD-9*] and *International Statistical Classification of Diseases and Related Health Problems, Tenth Revision* [*ICD-10*] diagnoses), laboratory test results, procedures (recorded by *Current Procedural Terminology* codes), pharmacy data, and mortality status. The study was approved by the Human Investigations Committee at the VHA Connecticut Healthcare System and Yale University and was granted a waiver of informed consent because this research could not practicably be conducted without a waiver of consent. This study followed the Strengthening the Reporting of Observational Studies in Epidemiology (STROBE) reporting guideline.^22^

### Study Cohort

Patients were included if they had (1) at least 1 outpatient visit in the VHA from January 1, 2014 (date after which DAAs were available within the system), through May 31, 2017 (to allow for up to 3 years of follow-up)^16^; (2) positive HCV antibody and/or detectable HCV RNA; and (3) a documented alcohol screening. Patients were excluded if they had undetectable HCV RNA throughout the study period with or without positive HCV antibody. Patients with positive HCV antibody with unknown HCV RNA status were included because laboratory studies may have been completed outside the VHA or barriers to confirmatory HCV RNA testing may have existed.

The index date was the date of the first outpatient visit or first positive HCV RNA and/or antibody from January 1, 2014, to May 31, 2017. The year prior to the index date served as the baseline period during which alcohol use, comorbidities, and laboratory test results were ascertained. Patients were followed up for up to 3 years from their index date, until May 31, 2020. The 2 primary outcomes were time to receipt of the initial DAA regimen (1) within 1 year after the index date because treatment should start as soon as possible and (2) within 3 years after the index date because the initial large volume of eligible patients may have affected timeliness of treatment.

### Data Collection

#### Alcohol Use Categories

Baseline alcohol use was categorized according to responses to the Alcohol Use Disorders Identification Test–Consumption (AUDIT-C) questionnaire^23,24,25,26^ and *ICD-9* or *ICD-10* diagnoses.^5^ The AUDIT-C questionnaire is a 3-item screening test, with scores ranging from 0 to 12 (where unhealthy alcohol use is signified by a score of 4 or more among men and 3 or more among women), that assesses past-year alcohol consumption as well as frequency of heavy episodic drinking (≥6 drinks per occasion).^23^ This instrument has good operating characteristics for the detection of at-risk drinking but is not sufficient to diagnose AUD.^27,28,29^ We additionally defined AUD using *ICD-9* or *ICD-10* inpatient or outpatient codes including alcohol abuse, alcohol dependence, or related conditions, such as alcohol-associated cardiomyopathy, liver disease, pancreatitis, gastritis, myopathy, and psychosis, which have been shown to identify current AUD.^30,31,32,33^

We created 5 mutually exclusive alcohol use categories: (1) current AUD (*ICD-9* or *ICD-10* AUD codes in the baseline year and nonzero AUDIT-C score); (2) at-risk drinking (AUDIT-C score ≥4 for men or ≥3 for women or reported consumption of ≥6 drinks on 1 occasion without the presence of *ICD-9* or *ICD-10* AUD codes); (3) lower-risk drinking (AUDIT-C score of 1-3 for men or 1-2 for women without the presence of *ICD-9* or *ICD-10* AUD codes); (4) abstinent with AUD history (presence of *ICD-9* or *ICD-10* AUD codes prior to the baseline year with an AUDIT-C score of 0); and (5) abstinent without AUD history (AUDIT-C score of 0 without the presence of *ICD-9* or *ICD-10* AUD codes). Among abstinent patients, we differentiated between those with and those without a history of AUD because the former may represent a distinct population consisting of those with AUD in remission, inability to tolerate alcohol owing to comorbidities, or inaccurate self-report.^32^

#### Demographic or Clinical Variables

Demographic and clinical data collected from the electronic health record included age; sex; race and ethnicity; body mass index; tobacco use; *ICD-9* or *ICD-10* diagnoses for HIV, hepatitis B virus (HBV) coinfection, cirrhosis, hepatic decompensation,^34^ hepatocellular carcinoma (HCC), liver transplant, diabetes, depression, severe mental illness (representing posttraumatic stress disorder, bipolar disorder, and/or schizophrenia), and history of drug use disorder (eTable in Supplement 1); laboratory test results including HCV genotype, alanine aminotransferase (ALT), aspartate aminotransferase (AST), and platelet count; and history of HCV treatment prior to 2014 with a non-DAA regimen. Platelet count, ALT level, and AST level were collected from dates closest to the index date within the window from up to 1 year prior to 30 days after. The Fibrosis-4 Index for Liver Fibrosis (FIB-4; based on 4 factors: age, AST level, ALT level, and platelet count), a noninvasive measure of hepatic fibrosis, was calculated.^35,36,37^ A FIB-4 score of more than 3.25 is associated with advanced hepatic fibrosis^35^ and may capture patients with compensated cirrhosis who may not otherwise be identified through diagnostic codes.^38,39^ Finally, we identified dispensed fills for DAA regimens, including boceprevir (with peginterferon alfa and ribavirin), elbasvir-grazoprevir, glecaprevir-pibrentasvir, ombitasvir-paritaprevir-ritonavir (with or without dasabuvir and/or ribavirin), ledipasvir-sofosbuvir (with or without ribavirin), sofosbuvir (with peginterferon alfa and/or ribavirin), sofosbuvir-daclatasvir (with or without ribavirin), sofosbuvir-simeprevir (with or without ribavirin), simeprevir (with peginterferon alfa and ribavirin), sofosbuvir-velpatasvir (with or without ribavirin and/or voxilaprevir), and telaprevir (with peginterferon alfa and ribavirin).

### Statistical Analysis

Demographic and clinical characteristics were described by alcohol use categories. We compared the percentage of patients with DAA treatment within 1 and 3 years of the index date by alcohol use for each calendar year using the χ^2^ test for trend. We estimated the association of alcohol use and other potential covariates with DAA treatment using Cox proportional hazards regression models. Models were stratified by year (2014 vs 2015-2017) because the challenge of treating the large number of patients newly eligible for DAAs in 2014 may have influenced access compared with subsequent years when relatively fewer patients became eligible. Univariate models were used to examine whether alcohol use and other potential covariates were associated with DAA treatment within 1 and 3 years. Patients were censored at DAA treatment date, death, or 1 year (for the 1-year models) or 3 years (for the 3-year models), whichever came first. Variables evaluated in univariate models included age, sex, race (collapsed to Black, White, and all other races [American Indian, Asian, mixed race, Pacific Islander, and unknown race] because of the low numbers of patients in categories other than Black or White), ethnicity (Hispanic or Latinx or non-Hispanic or Latinx), severity of liver disease (FIB-4 score, cirrhosis, hepatic decompensation, HCC, or liver transplant), comorbidities (HBV, HIV, diabetes, drug use disorder, depression, or severe mental illness), prior HCV treatment, and year of cohort entry. These variables, including race and ethnicity, were included because of their potential to be associated with DAA treatment. Variables statistically significantly associated with DAA treatment in univariate models (*P* < .05) were included in multivariable models. Unadjusted and adjusted hazard ratios (HRs) with 95% CIs were estimated using lower-risk drinking as the reference category because patients in abstinent categories likely represent a heterogeneous group, including those with the inability to tolerate alcohol owing to comorbidities, inaccurate self-report, or AUD in remission.^32^ Analyses were run using Stata, version 14.0 (StataCorp LLC). All *P* values were from 2-sided tests, and results were deemed statistically significant at *P* < .05.

## Results

### Demographic and Clinical Characteristics

We identified 133 753 patients eligible for DAAs during 2014-2017. Patients had a mean (SD) age of 60.6 (4.5) years; were primarily male (97%), White (55%), or Black (40%); and had primarily HCV genotype 1 (67%) (Table 1). Twenty-two percent of patients had a FIB-4 score higher than 3.25, 12% had cirrhosis, and 4% had hepatic decompensation. Forty-seven percent of patients had a drug use disorder, 34% had depression, and 36% had severe mental illness. Thirty-eight percent of patients had current AUD, 6% had at-risk drinking, 14% had lower-risk drinking, 12% were abstinent with a history of AUD, and 30% were abstinent without a history of AUD. Most patients became eligible to receive DAA treatment in 2014 (86%), with 7% eligible in 2015, 5% eligible in 2016, and 2% eligible in 2017.

**Table 1.** Characteristics, by Alcohol Use Category, of Patients Eligible for HCV Treatment Within the Veterans Health Administration Birth Cohort From January 1, 2014, to May 31, 2017

Baseline characteristic	Patients, No. (%)					
	Overall (N = 133 753)	Current AUD (n = 51 372)	At-risk drinking (n = 7551)	Lower-risk drinking (n = 18 230)	Abstinent with AUD history (n = 16 063)	Abstinent without AUD history (n = 40 537)
Age, mean (SD), y	60.6 (4.5)	59.9 (4.5)	60.7 (4.5)	60.9 (4.5)	60.7 (4.4)	61.3 (4.4)
Sex						
Female	3650 (3)	1051 (2)	224 (3)	629 (4)	363 (2)	1383 (3)
Male	130 103 (97)	50 321 (98)	7327 (97)	17 601 (97)	15 700 (98)	39 154 (97)
Race						
American Indian	1055 (1)	411 (1)	70 (1)	123 (1)	121 (1)	330 (1)
Asian	246 (0.2)	60 (0.1)	10 (0.1)	39 (0.2)	22 (0.1)	115 (0.3)
Black	53 032 (40)	22 225 (43)	2301 (31)	6664 (37)	7219 (45)	14 623 (36)
Mixed race	857 (1)	332 (1)	39 (1)	118 (1)	100 (1)	268 (1)
Pacific Islander	516 (0.4)	159 (0.3)	47 (1)	77 (0.4)	40 (0.3)	193 (1)
White	73 493 (55)	26 878 (52)	4660 (62)	10 388 (57)	8251 (51)	23 316 (58)
Unknown	4554 (3)	1307 (3)	424 (6)	821 (5)	310 (2)	1692 (4)
Ethnicity						
Hispanic or Latinx	7266 (5)	2655 (5)	436 (6)	912 (5)	867 (5)	2396 (6)
Non-Hispanic or Latinx	126 487 (95)	48 717 (95)	7114 (94)	17 318 (95)	15 196 (95)	38 141 (94)
FIB-4 score						
<1.45	31 438 (24)	11 123 (22)	1762 (23)	4829 (27)	3876 (24)	9848 (24)
1.45-3.25	55 604 (42)	20 568 (40)	3126 (41)	7928 (44)	6562 (41)	17 420 (43)
>3.25	29 769 (22)	13 432 (26)	1666 (22)	3068 (17)	3262 (20)	8341 (21)
Unknown or missing	16 942 (13)	6249 (12)	997 (13)	2405 (13)	2363 (15)	4925 (12)
BMI category						
Underweight	2546 (2)	1189 (2)	143 (2)	278 (2)	305 (2)	631 (2)
Normal	41 283 (31)	18 648 (36)	2470 (33)	5041 (28)	4582 (29)	10 542 (26)
Overweight	48 425 (36)	17 970 (35)	2677 (36)	6887 (38)	5783 (36)	15 077 (37)
Obesity Class I	26 078 (20)	8522 (17)	1444 (19)	3828 (21)	3375 (21)	8909 (22)
Obesity Class II	8844 (7)	2629 (5)	489 (7)	1359 (8)	1175 (7)	3192 (8)
Obesity Class III	3751 (3)	966 (2)	189 (3)	552 (3)	476 (3)	1571 (4)
Unknown or missing	2826 (2)	1448 (3)	108 (1)	289 (2)	367 (2)	615 (2)
HCV genotype						
1	90 177 (67)	35 625 (69)	4429 (59)	11 560 (63)	11 632 (72)	26 931 (66)
2	10 116 (8)	3781 (7)	617 (8)	1407 (8)	1216 (8)	3095 (8)
3	6357 (5)	2580 (5)	348 (5)	745 (4)	835 (5)	1849 (5)
4	1039 (1)	419 (1)	46 (1)	133 (1)	124 (1)	317 (1)
5	8 (0.006)	3 (0.006)	0	0	1 (0.006)	4 (0.01)
6	12 (0.009)	4 (0.008)	0	2 (0.01)	1 (0.006)	5 (0.01)
Multiple	401 (0.3)	183 (0.4)	12 (0.2)	49 (0.3)	70 (0.4)	87 (0.2)
Unknown or missing	25 643 (19)	8777 (17)	2099 (28)	4334 (24)	2184 (14)	8249 (20)
Prior treatment	9939 (7)	3124 (6)	278 (4)	1006 (6)	1693 (11)	3838 (10)
Comorbidity						
HIV	3651 (3)	1487 (3)	114 (2)	396 (2)	612 (4)	1042 (3)
HBV	7689 (6)	3801 (7)	200 (3)	566 (3)	1463 (9)	1659 (4)
Diabetes	35 707 (27)	11 983 (23)	1197 (16)	4436 (24)	5411 (34)	12 680 (31)
Drug use disorder	62 391 (47)	36 319 (71)	1023 (14)	3281 (18)	12 547 (78)	9221 (23)
Depression	45 340 (34)	23 254 (45)	811 (11)	3040 (17)	8492 (53)	9743 (24)
Severe mental illness	48 093 (36)	23 668 (46)	964 (13)	3345 (18)	8965 (56)	11 151 (28)
Hepatic decompensation	4968 (4)	2515 (5)	51 (1)	150 (1)	891 (6)	1361 (3)
HCC	2418 (2)	1073 (2)	32 (0.4)	107 (1)	417 (3)	789 (2)
Liver transplant	1143 (1)	175 (0.3)	6 (0.1)	43 (0.2)	254 (2)	665 (2)
Cirrhosis	15 988 (12)	7344 (14)	274 (4)	949 (5)	2574 (16)	4847 (12)

Abbreviations: AUD, alcohol use disorder; BMI, body mass index; FIB-4, fibrosis index based on 4 factors (age, aspartate aminotransferase level, alanine aminotransferase level, and platelet count); HBV, hepatitis B virus; HCC, hepatocellular carcinoma; HCV, hepatitis C virus.

### Receipt of DAA Treatment by Year and Alcohol Use Category

For patients entering the cohort in 2014, 2015, 2016, and 2017, the receipt of DAA treatment within 1 year of eligibility was 7%, 33%, 53%, and 56%, respectively, and the receipt of DAA treatment within 3 years was 47%, 58%, 65%, and 65%, respectively (Figure). In 2014, 5% of those with current AUD received DAAs within 1 year compared with 8% to 10% of those in other alcohol use categories. In 2017, 50% of those with current AUD and 49% of those with past AUD received DAAs within 1 year compared with 57% to 60% of those in other alcohol use categories. The pattern was similar for receipt of DAA treatment within 3 years (Figure). The associations between DAA treatment receipt (within 1 and 3 years) and alcohol use were statistically significant based on the χ^2^ test for trend (*P* < .001 for all associations except *P* = .005 for DAA treatment within 3 years among those eligible in 2017; for DAA treatment within 1 year, χ^2^ tests for trend values were 625.9, 126.1, 53.6, and 12.2 for years 2014, 2015, 2016, and 2017, respectively; for DAA treatment within 3 years, χ^2^ tests for trend values were 969.6, 94.7, 30.0, and 7.8 for years 2014, 2015, 2016, and 2017, respectively).

**Figure.**
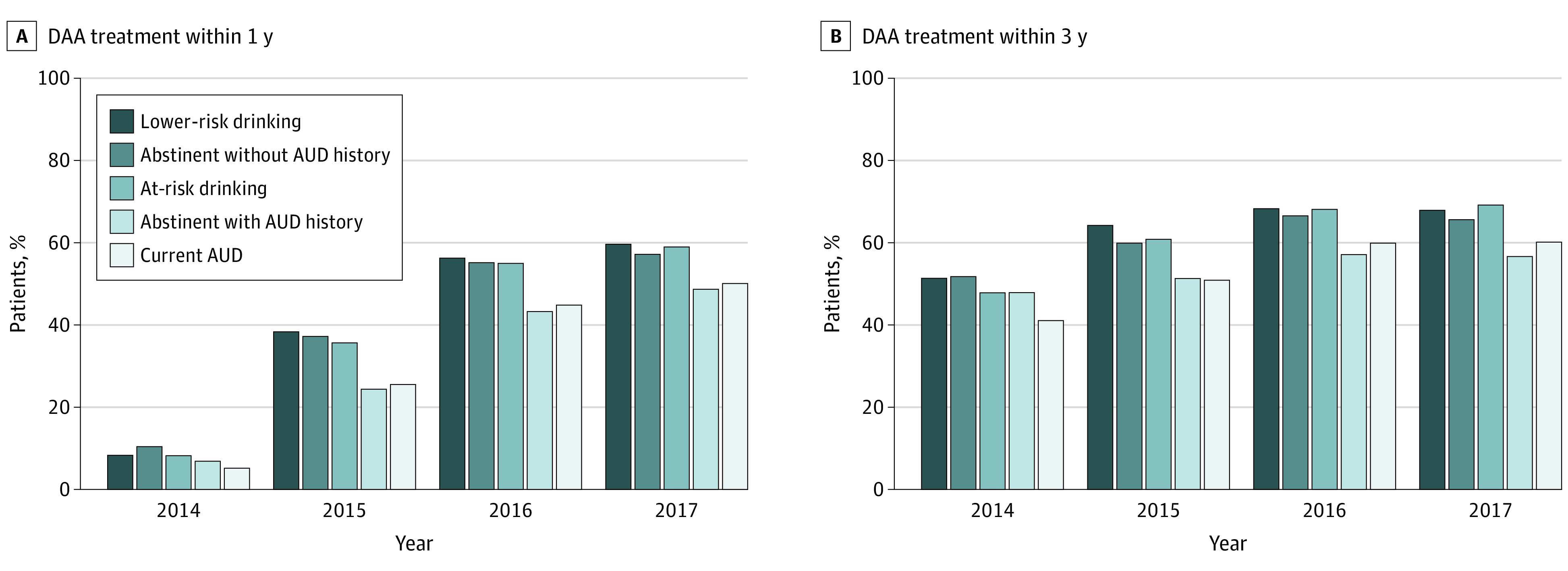
Percentage of Patients With Hepatitis C Virus Within Veterans Health Administration Birth Cohort Receiving Direct-Acting Antiviral (DAA) Treatment Within 1 and 3 Years by Alcohol Use Category and Index Year AUD indicates alcohol use disorder.

### Association Between Alcohol Use and Receipt of DAA Treatment, 2014

For patients eligible for DAAs in 2014, based on adjusted models, those who were abstinent without a history of AUD were more likely (HR, 1.10 [95% CI, 1.03-1.18]) to receive DAAs within 1 year compared with patients with lower-risk drinking (Table 2). Those with current AUD (HR, 0.72 [95% CI, 0.66-0.77]) or abstinent with an AUD history (HR, 0.91 [95% CI, 0.84-1.00]) were less likely to recieve DAAs within 1 year compared with patients with lower-risk drinking. Those with at-risk alcohol use (HR, 0.93 [95% CI, 0.89-0.98]) and current AUD (HR, 0.81 [95% CI, 0.79-0.83]) were less likely to receive DAAs within 3 years than those with lower-risk drinking. Additional information on the association of covariates with DAA treatment is shown in Table 2.

**Table 2.** Adjusted Hazard Ratios for Receipt of DAA Treatment Within 1 and 3 Years Among Patients Eligible for HCV Treatment Within the Veterans Health Administration Birth Cohort^a^

Characteristic	Hazard ratio (95% CI)			
	DAA treatment within 1 y		DAA treatment within 3 y	
	2014 (n = 115 272)	2015-2017 (n = 18 481)	2014 (n = 115 272)	2015-2017 (n = 18 481)
Alcohol use				
Lower-risk drinking	1 [Reference]	1 [Reference]	1 [Reference]	1 [Reference]
Abstinent without AUD history	1.10 (1.03-1.18)	0.98 (0.93-1.04)	1.01 (0.99-1.04)	0.97 (0.93-1.02)
At-risk drinking	0.96 (0.86-1.08)	0.94 (0.87-1.02)	0.93 (0.89-0.98)	0.96 (0.90-1.03)
Abstinent with AUD history	0.91 (0.84-1.00)	0.76 (0.68-0.86)	0.99 (0.95-1.02)	0.81 (0.73-0.89)
Current AUD	0.72 (0.66-0.77)	0.75 (0.70-0.81)	0.81 (0.79-0.83)	0.80 (0.76-0.85)
Sex				
Female	1.22 (1.08-1.37)	1.16 (1.02-1.32)	1.05 (1.00-1.11)	1.14 (1.02-1.27)
Male	1 [Reference]	1 [Reference]	1 [Reference]	1 [Reference]
Age, y	0.98 (0.98-0.99)	1.00 (1.00-1.01)	1.00 (1.00-1.01)	1.00 (1.00-1.00)
Race and ethnicity				
Black	0.69 (0.66-0.73)	0.81 (0.77-0.85)	0.89 (0.87-0.91)	0.88 (0.85-0.92)
Hispanic or Latinx	0.70 (0.61-0.81)	0.88 (0.71-1.10)	0.92 (0.87-0.97)	0.90 (0.75-1.08)
White	1 [Reference]	1 [Reference]	1 [Reference]	1 [Reference]
Other	0.88 (0.80-0.97)	0.84 (0.77-0.92)	0.90 (0.86-0.93)	0.86 (0.80-0.92)
Prior HCV treatment	3.69 (3.51-3.87)	0.68 (0.54-0.88)	1.72 (1.67-1.76)	0.72 (0.59-0.87)
Diabetes	0.75 (0.71-0.79)	0.94 (0.88-1.01)	0.88 (0.86-0.90)	0.94 (0.89-0.99)
BMI category				
Normal	1 [Reference]	1 [Reference]	1 [Reference]	1 [Reference]
Underweight	0.46 (0.35-0.62)	0.80 (0.67-0.95)	0.74 (0.68-0.81)	0.79 (0.69-0.91)
Overweight or obesity	1.31 (1.25-1.38)	1.18 (1.12-1.24)	1.24 (1.22-1.27)	1.14 (1.09-1.18)
Missing	0.56 (0.42-0.75)	0.73 (0.61-0.86)	0.75 (0.69-0.82)	0.71 (0.62-0.82)
HIV	1.07 (0.94-1.21)	0.61 (0.47-0.79)	1.19 (1.14-1.25)	0.58 (0.47-0.72)
HBV	0.85 (0.77-0.94)	0.98 (0.86-1.13)	0.93 (0.90-0.97)	0.88 (0.55-1.11)
Drug use disorder	0.71 (0.67-0.75)	0.76 (0.72-0.82)	0.78 (0.76-0.80)	0.81 (0.77-0.86)
Depression	0.81 (0.68-0.75)	1.00 (0.92-1.08)	0.99 (0.97-1.01)	0.99 (0.93-1.05)
Severe mental illness	0.87 (0.77-0.85)	0.87 (0.81-0.94)	0.94 (0.92-0.96)	0.91 (0.86-0.97)
Liver transplant	1.43 (1.25-1.63)	1.59 (1.09-2.33)	1.31 (1.21-1.42)	1.47 (1.03-2.11)
Cirrhosis	1.51 (1.43-1.59)	0.96 (0.82-1.12)	1.14 (1.11-1.17)	0.83 (0.73-0.95)
HCV genotype				
1	1 [Reference]	1 [Reference]	1 [Reference]	1 [Reference]
2	1.65 (1.54-1.76)	0.95 (0.88-1.03)	0.85 (0.83-0.88)	0.98 (0.91-1.05)
3	1.08 (0.99-1.18)	0.70 (0.63-0.78)	0.78 (0.75-0.81)	0.74 (0.68-0.81)
4	0.71 (0.55-0.93)	0.91 (0.68-1.20)	0.82 (0.75-0.90)	0.89 (0.70-1.14)
Other or multiple	1.27 (0.93-1.74)	0.84 (0.45-1.57)	1.12 (0.99-1.28)	0.98 (0.60-1.61)
Unknown	0.17 (0.15-0.20)	0.20 (0.19-0.22)	0.08 (0.07-0.08)	0.24 (0.23-0.25)
FIB-4 score				
1.45-3.25	1 [Reference]	1 [Reference]	1 [Reference]	1 [Reference]
<1.45	0.71 (0.66-0.76)	0.89 (0.84-0.94)	0.88 (0.86-0.90)	0.88 (0.84-0.92)
>3.25	1.81 (1.72-1.90)	0.98 (0.92-1.04)	1.19 (1.16-1.22)	0.97 (0.92-1.02)
Unknown	0.65 (0.59-0.71)	0.59 (0.54-0.64)	0.78 (0.75-0.80)	0.66 (0.62-0.71)
Index year				
2015	1 [Reference]	1 [Reference]	1 [Reference]	1 [Reference]
2016	NA	2.31 (2.20-2.43)	NA	1.68 (1.62-1.75)
2017	NA	6.49 (6.00-7.01)	NA	3.66 (3.43-3.90)

Abbreviations: AUD, alcohol use disorder; BMI, body mass index; DAA, direct-acting antiviral; FIB-4, fibrosis index based on 4 factors (age, aspartate aminotransferase level, alanine aminotransferase level, and platelet count); HBV, hepatitis B virus; HCC, hepatocellular carcinoma; HCV, hepatitis C virus; NA, not applicable.

^a^ Adjusted for age, sex, race, ethnicity, diabetes, HIV, HBV, drug use disorder, depression, severe mental illness, liver transplant, hepatic decompensation, HCC, HCV genotype, FIB-4 score, prior treatment for HCV, and time entering cohort (index time).

### Association Between Alcohol Use and Receipt of DAA Treatment, 2015-2017

For patients eligible for DAAs in 2015-2017, in adjusted models, those with current AUD (HR, 0.75 [95% CI, 0.70-0.81]) or abstinent with an AUD history (HR, 0.76 [95% CI, 0.68-0.86]) were less likely to receive DAAs within 1 year compared with those with lower-risk drinking (Table 2). Similarly, those with current AUD (HR, 0.80 [95% CI, 0.76-0.85]) or abstinent with an AUD history (HR, 0.81 [95% CI, 0.73-0.89]) were less likely to recieve DAAs within 3 years compared with those with lower-risk drinking. Additional information on the association of covariates with DAA treatment is shown in Table 2.

## Discussion

Among patients with HCV receiving care in the VHA during the DAA era, individuals with AUD were less likely to receive DAAs compared with those with lower-risk drinking. This association persisted within both the 2014 and 2015-2017 periods and held true for DAA treatment at 1 and 3 years.

There are several possible mechanisms that may explain the lower likelihood of DAA treatment among patients with HCV and AUD. Despite guideline recommendations, health care professionals may assume that patients with AUD or other SUDs will be nonadherent to DAA treatment, which may influence their decision to place a referral or initiate DAA treatment.^40^ For instance, 1 study^41^ indicated that less than 60% of clinicians were willing to prescribe DAAs for patients currently drinking alcohol. Clinicians were also reluctant to refer patients for HCV treatment if there were mental health concerns or substance use.^40^ In addition, patient-level factors, such as competing priorities, fear of adverse effects, stigma, and negative interactions with health care professionals, may be associated with acceptance of DAAs or referrals for specialty care.^42,43^ Finally, systemwide factors associated with the lower likelihood of DAA treatment among patients with HCV and AUD may also exist. Outside the VHA, the high cost associated with DAA treatment has led to payer-associated barriers in the form of prior authorizations and policies requiring abstinence from alcohol and other substances to be eligible for insurance coverage.^44,45^ Although insurance coverage is not a barrier for patients receiving care within the VHA, it took several years after the availability of DAAs for sufficient funds to be secured to treat all patients with HCV^13^; it is possible that clinicians may have initially adopted similar practices with regard to alcohol and other substances when triaging patients for DAA treatment. Consistent with AASLD-IDSA recommendations, VHA HCV treatment guidelines also indicate that alcohol use, substance use, and mental illness are not absolute contraindications to DAA treatment; however, clinicians are advised to consider treating such patients on a case-by-case basis and coordinate care with mental health and addiction specialists.^15,16,17^ Although facilitating such treatment is an important aspect of providing holistic care for patients with HCV and AUD or other SUDs, an unintended consequence may have been additional delays affecting the HCV cascade of care for this group.^4,46^

Disparities in the receipt of DAA treatment for patients with AUD persisted for several years after the availability of DAAs despite extensive efforts to eradicate HCV within the VHA.^13^ This disparity is concerning because of the importance of timely DAA treatment for those at increased risk of hepatic fibrosis^1^ and because patients with unhealthy alcohol use and HCV can achieve high rates of SVR.^12^ Furthermore, there seem to be disparities in the receipt of DAA treatment even among those with self-reported abstinence but with a history of AUD. Finally, lower levels of DAA treatment among patients with severe mental illness and other drug use disorders highlight the need for further research to evaluate the factors associated with HCV treatment as well as interventions to improve access in these subgroups.

The associations of race other than White and Hispanic or Latinx ethnicity with lower receipt of DAA treatment are consistent with prior literature on racial and ethnic disparities in HCV treatment and emphasize the need for culturally appropriate and antiracist efforts to improve equity in care.^47,48,49^ Black patients and patients from other minoritized racial and ethnic groups were less likely to receive DAAs at 1 and 3 years throughout the study period. Patients of Hispanic or Latinx ethnicity who entered the cohort in 2014 were less likely to receive DAAs within 1 and 3 years; however, this trend did not persist in 2015-2017. Racial and ethnic disparities in DAA treatment are particularly troubling because Black and Hispanic or Latinx patients are at greater risk of presenting with HCC and are less likely to receive a liver transplant for end-stage liver disease.^50,51^ Further research to explore the structural determinants of HCV treatment for patients of minoritized racial and ethnic groups is needed.

### Strengths and Limitations

This study has several strengths. The population consisted of a large national cohort that was screened for HCV in a system providing universal DAA coverage. Furthermore, adjustment for covariates, including psychiatric conditions, drug use disorders, and liver fibrosis, demonstrated that the association between AUD and lower receipt of DAA treatment was independent of these factors.

This study also has some limitations. First, alcohol use categories were based on self-report and *ICD-9* or *ICD-10* diagnoses, which may be subject to bias or misclassification.^52,53^ For instance, individuals with high AUDIT-C scores without *ICD-9* or *ICD-10* codes for AUD may have had current AUD, although a small number of patients in our study fit this profile. Social desirability bias might also result in decreased self-report of alcohol consumption among patients with HCV. Self-report of alcohol use has been validated and remains the standard for research and medication approvals, although biomarkers may increase accuracy.^54,55,56,57^ Second, we could not determine the reason a patient did not receive DAA treatment and whether the prescriber did not offer it or the patient refused treatment. Because we measured DAA treatment in terms of filled prescriptions, we did not include prescriptions written but not filled. Patients may also have received DAA treatment outside of the VHA. In addition, factors such as estimated life expectancy, which could be associated with DAA prescribing, were not examined. Third, we did not examine rates of SVR, although prior research demonstrated that patients with unhealthy alcohol use can achieve similar SVR rates compared with those who are abstinent or consume lower levels of alcohol.^12^ Fourth, practice changes that may have occurred after 2020 with regard to facilitating DAA treatment for groups experiencing barriers, such as patients with AUD, would not be reflected in this analysis. Fifth, our results may not be fully generalizable outside the VHA system.

## Conclusions

The findings of this cohort study should be considered in light of prior work^5^ demonstrating a high prevalence of advanced hepatic fibrosis and cirrhosis among individuals who drink alcohol at levels thought to be “safe” and literature^7^ indicating poorer outcomes among patients with unhealthy alcohol use after DAA treatment. Given the higher risk of liver-related complications, DAA treatment should be prioritized for patients with HCV and unhealthy alcohol use. The VHA has used implementation strategies to support HCV treatment using clinical teams and patient outreach.^14,58^ Similar efforts should be made to address disparities in DAA treatment using integrated care models that address unhealthy alcohol use and HCV treatment simultaneously. In the context of clinical trials, analogous stepped treatment models have been implemented in HIV clinics to reach individuals with HIV and AUD.^59^ Providing alcohol treatment in liver clinics can decrease alcohol consumption among patients with HCV as well.^60,61,62^ Further research to determine barriers to and facilitators of DAA treatment for patients with HCV and AUD and to develop interventions to ensure access to DAAs are needed.

## Notes

Supplement 1. **eTable.** *ICD* Codes Used to Define Study Variables

Supplement 2.
Data Sharing Statement
