# Error in Byline

**DOI:** 10.1001/jamanetworkopen.2022.53120

**Published:** 2023-01-09

In the Original Investigation titled “Association Between Alcohol Use Disorder and Receipt of Direct-Acting Antiviral Hepatitis C Virus Treatment,”^1^ published December 14, 2022, the academic degree shown in the byline for Dr Tate should have been MPH, ScD rather than PhD. This article has been corrected.^1^
