# Supplementary material for: Association Between Alcohol Use Disorder and Receipt of Direct-Acting Antiviral Hepatitis C Virus Treatment
Source: JAMA Netw Open. 2022 Dec 14;5(12):e2246604. doi: 10.1001/jamanetworkopen.2022.46604 (PMC9856353; doi:10.1001/jamanetworkopen.2022.46604)
Supplement: Supplement 1. — eTable. ICD Codes Used to Define Study Variables [file jamanetwopen-e2246604-s001.pdf]

Supplementary Online Content

Haque LY, Fiellin DA, Tate JP, et al. Association between alcohol use disorder and receipt of direct-acting antiviral hepatitis C virus treatment. *JAMA Netw Open.* 2022;5(12):e2246604. doi:10.1001/jamanetworkopen.2022.46604

eTable. *ICD* Codes Used to Define Study Variables

This supplementary material has been provided by the authors to give readers additional information about their work.

© 2022 Haque LY et al. *JAMA Network Open.*

eTable. *ICD* Codes Used to Define Study Variables

Condition	*ICD* Codes**
Cirrhosis	571.2, 571.5, 571.6
Hepatic decompensation	456.0, 456.20, 567.23, 572.2, 572.4, 789.5, 789.59
Hepatocellular cancer	155.0
Liver transplant	996.82, V42.7
Alcohol use disorder	303.00, 303.01, 303.02, 303.03, 303.90, 303.91, 303.92, 303.93, 305.00, 305.01, 305.02,305.03, F10.229, F10.20, F10.21, F10.10
Drug use disorder	304.2, 304.0, 304.7, 304.3, 304.1, 304.4, 304.5, 304.6, 304.8, 304.9, 305.6, 305.5, 305.2, 305.3, 305.4, 305.7, 305.8, 305.9, 292.x, except 304.23, 304.03, 304.73, 304.33, 304.13, 304.43, 304.53, 304.63, 304.83, 304.93, 305.63, 305.53, 305.23, 305.33, 305.43, 305.73, 305.83, 305.93
Human immunodeficiency virus (HIV)	042,V08, B20, Z21
Diabetes	249.00, 250.00, 250.01, 249.01, 249.10, 249.11, 249.20, 249.21, 249.30, 249.31, 249.40, 249.41, 249.50, 249.51, 249.60, 249.61, 249.70, 249.71, 249.80, 249.81, 249.90, 249.91, 250.02, 250.03, 250.10, 250.11, 250.12, 250.13, 250.20, 250.21, 250.22, 250.23, 250.30, 250.31, 250.32, 250.33, 250.40, 250.41, 250.42, 250.43, 250.50, 250.51, 250.52, 250.53, 250.60, 250.61, 250.62, 250.63, 250.70, 250.71, 250.72, 250.73, 250.80, 250.81, 250.82, 250.83, 250.90, 250.91, 250.92, 250.93, 357.2
Post-traumatic stress disorder	309.81
Bipolar disorder	296.0, 296.00, 296.01, 296.02, 296.03, 296.04, 296.05, 296.06, 296.1, 296.10, 296.11, 296.12, 296.13, 296.14, 296.15, 296.16, 296.4, 296.40, 296.41, 296.42, 296.43, 296.44, 296.45, 296.46, 296.5, 296.50, 296.51, 296.52, 296.53, 296.54, 296.55, 296.56, 296.6, 296.60, 296.61, 296.62, 296.63, 296.64, 296.65, 296.66, 296.7, 296.70, 296.71, 296.72, 296.73, 296.74, 296.75, 296.76, 296.8, 296.80, 296.81, 296.82, 296.89, 296.9, 296.90, 296.99
Schizophrenia	295.x
Depression	296.20, 296.21, 296.22, 296.23, 296.24, 296.25, 296.26, 296.30, 296.31, 296.32, 296.33, 296.34, 296.35, 296.36, 293.83, 296.90, 296.99, 300.4, 309.0, 309.1, 311

Note: Severe mental illness was defined as having at least one of the following chronic conditions: post-traumatic stress disorder; bipolar disorder; schizophrenia

© 2022 Haque LY et al. *JAMA Network Open.*
