# Supplementary material for: Association Between Alcohol Use Disorder and Receipt of Direct-Acting Antiviral Hepatitis C Virus Treatment
Source: JAMA Netw Open. 2022 Dec 14;5(12):e2246604. doi: 10.1001/jamanetworkopen.2022.46604 (PMC9856353; doi:10.1001/jamanetworkopen.2022.46604)
Supplement: Supplement 2. — Data Sharing Statement [file jamanetwopen-e2246604-s002.pdf]

Data Sharing Statement

Haque. Association Between Alcohol Use Disorder and Receipt of Direct-Acting Antiviral Hepatitis C Virus Treatment. *JAMA Netw Open*. Published December 14, 2022. doi:10.1001/jamanetworkopen.2022.46604

Data
Data available: Yes
Data types: Deidentified participant data
How to access data: Due to US Department of Veterans Affairs (VA) regulations and our ethics agreements, the analytic data sets used for this study are not permitted to leave the VA firewall without a Data Use Agreement. This limitation is consistent with other studies based on VA data. However, VA data are made freely available to researchers with an approved VA study protocol. For more information, please visit https://www.virec.research.va.gov.
When available: With publication

Supporting Documents
Document types: None

Additional Information
Who can access the data: Due to US Department of Veterans Affairs (VA) regulations and our ethics agreements, the analytic data sets used for this study are not permitted to leave the VA firewall without a Data Use Agreement. This limitation is consistent with other studies based on VA data. However, VA data are made freely available to researchers with an approved VA study protocol. For more information, please visit https://www.virec.research.va.gov.
Types of analyses: Due to US Department of Veterans Affairs (VA) regulations and our ethics agreements, the analytic data sets used for this study are not permitted to leave the VA firewall without a Data Use Agreement. This limitation is consistent with other studies based on VA data. However, VA data are made freely available to researchers with an approved VA study protocol. For more information, please visit https://www.virec.research.va.gov.
Mechanisms of data availability: Due to US Department of Veterans Affairs (VA) regulations and our ethics agreements, the analytic data sets used for this study are not permitted to leave the VA firewall without a Data Use Agreement. This limitation is consistent with other studies based on VA data. However, VA data are made freely available to researchers with an approved VA study protocol. For more information, please visit https://www.virec.research.va.gov.
